# Comparative genomics of vesicomyid clam (Bivalvia: Mollusca) chemosynthetic symbionts

**DOI:** 10.1186/1471-2164-9-585

**Published:** 2008-12-04

**Authors:** Irene LG Newton, Peter R Girguis, Colleen M Cavanaugh

**Affiliations:** 1 Harvard University, Organismic and Evolutionary Biology, 16 Divinity Avenue, Cambridge, MA 02138, USA; 2 Department of Microbiology, Tufts University, 136 Harrison Avenue, Boston, MA 02111, USA

## Abstract

**Background:**

The Vesicomyidae (Bivalvia: Mollusca) are a family of clams that form symbioses with chemosynthetic gamma-proteobacteria. They exist in environments such as hydrothermal vents and cold seeps and have a reduced gut and feeding groove, indicating a large dependence on their endosymbionts for nutrition. Recently, two vesicomyid symbiont genomes were sequenced, illuminating the possible nutritional contributions of the symbiont to the host and making genome-wide evolutionary analyses possible.

**Results:**

To examine the genomic evolution of the vesicomyid symbionts, a comparative genomics framework, including the existing genomic data combined with heterologous microarray hybridization results, was used to analyze conserved gene content in four vesicomyid symbiont genomes. These four symbionts were chosen to include a broad phylogenetic sampling of the vesicomyid symbionts and represent distinct chemosynthetic environments: cold seeps and hydrothermal vents.

**Conclusion:**

The results of this comparative genomics analysis emphasize the importance of the symbionts' chemoautotrophic metabolism within their hosts. The fact that these symbionts appear to be metabolically capable autotrophs underscores the extent to which the host depends on them for nutrition and reveals the key to invertebrate colonization of these challenging environments.

## Background

Symbiosis between prokaryotic and eukaryotic cells is a globally important phenomenon that influences the physiology, ecology, and evolution of virtually every organism on this planet [1-3]. Eukaryotic hosts expand their ecological niches through symbiosis with these metabolically diverse bacteria and archaea. An illustrative case is that of the chemosynthetic endosymbionts, which enable their hosts to populate and thrive in challenging environments such as deep-sea hydrothermal vents and cold seeps [4]. In these environments, reduced inorganic compounds are generated either biotically (e.g. microbial sulfate reduction) or abiotically (e.g. hydrothermal alteration). Chemosynthetic symbionts use the energy derived from the oxidation of these molecules to fix inorganic carbon [5]. Benefits for both partners in chemosynthetic symbioses are evident. The bacteria gain further access to the energy substrates they require from both oxic and anoxic habitats while the animals are provided with much, if not all, of their nutritional requirements [6-8]. The intimate structural and metabolic coupling often found in chemosynthetic symbioses underscores the importance of these relationships to host survival.

The vesicomyid clams are one of the better studied chemosynthetic symbioses and exist at hydrothermal vents, hydrocarbon seeps, and other chemically reduced environments. They are also relatively young as a group, as vesicomyid fossils date the formation of the symbiosis to the Cretaceous, between 50–100 Ma ago [9]. These clams have a greatly reduced gut and feeding groove [10] and, based on isotopic evidence, are thought to depend almost entirely on their endosymbionts for their carbon [11-13]. With respect to the animal host, the association is essential – no living vesicomyids have been found devoid of symbionts. Furthermore, these symbionts have not yet been found outside the host, have never been cultured in the laboratory, and are thought to be predominantly maternally transmitted each generation via the egg [14-16].

Previous studies of other bacterial symbionts suggest that symbiont transmission strategy is a predominant factor governing nutritional symbiont genome evolution. Bacterial symbionts transferred to the next host generation via the egg (vertical transmission) experience population bottlenecks upon transmission and few opportunities for recombination [17]. Because of the underlying deletion bias in bacterial genome evolution, and the limited amount of gene flow available to these symbionts, their genomes are minimized. For example, the genomes of the vertically transmitted, mutualistic insect endosymbionts *Buchnera*, *Baumannia*, *Blochmannia*, and *Wigglesworthia *are all reduced in size and content [18-21], exhibiting few chromosomal rearrangements, or horizontal gene transfer events [22-25]. However, these insect nutritional symbionts retain the genomic repetoire needed to provide key metabolic intermediates, vitamins, and amino acids often missing from their hosts' specialized diets [26], suggesting that host nutritional needs might select for retention of specific biosynthetic pathways. Conversely, those pathways redundant with host capabilities or nutrition are often lost completely [26]. In contrast to the strictly vertically transmitted symbionts, those that undergo occasional environmental or horizontal transmission (lateral acquisition) tend to have slightly larger genomes that exhibit evidence of recombination. For example, *Wolbachia pipientis*, the ubiquitous insect reproductive parasite, may be laterally transmitted [27,28], and their genomes are littered with mobile genetic elements, prophages and harbor clear evidence of past recombination events [29,30].

It must be noted, however, that transmission strategy for many symbionts cannot be distinctly or clearly demarcated; depending on the association, symbionts are perpetuated via a spectrum from strict vertical transmission to lateral acquisition. The insect reproductive parasites (such as *Wolbachia *and CFBs) are vertically transmitted but occasionally laterally acquired. Indeed, there is also some phylogenetic evidence, in the form of incongruent host and symbiont trees, against strict vertical transmission of the vesicomyid symbionts [31]. More recently, two different strains of the vesicomyid symbionts have been found within the same host, corroborating the lateral acquisition hypothesis suggested by the phylogenetic evidence above [32]. However, the vesicomyid symbionts are found in host primary oocytes [15,16] and their genomes are reduced, and exhibit a high A+T content [33,34], suggesting that although occasional lateral transmission may occur, the predominant transmission strategy used by the vesicomyids is vertical.

This mixed transmission strategy suggested for the vesicomyid symbionts has the potential to influence the genomic evolution of these bacteria. Research on the comparative evolutionary genomics of insect symbionts [18,19,21,35,36] suggests that symbionts with strictly vertical transmission strategies would lose genetic material redundant with host capabilities and retain metabolic pathways necessary for host survival. Occasional lateral transmission might offer the opportunity for recombination and horizontal gene transfer, possibly mitigating the negative effects associated with a reduction in population size. Indeed, some evidence of recombination has been found in the vesicomyid symbiont genomes, suggesting that genomic evolution of these bacteria may not be as straightforward as in strictly vertically transmitted symbionts [32].

We chose a comparative framework, utilizing both genomic and heterologous microarray data, to investigate genome evolution in the vesicomyid chemosynthetic symbionts. The genomes of the two fully sequenced vesicomyid symbionts, *Ruthia magnifica*, isolated from hydrothermal vents, and *Vesicomyosocious okutanii*, isolated from cold seeps [33,34] were compared to each other and to that of *Thiomicrospira crunogena*, a free-living chemoautotroph isolated from hydrothermal vents and the closest sequenced relative of the vesicomyid symbionts [37,38]. The availability of sequenced vesicomyid symbiont genomic data allowed us to develop microarrays for genome-scale analyses of conserved gene content in other vesicomyid symbionts. Indeed, the great amount of genetic conservation and synteny between the two sequenced vesicomyid symbiont genomes [39] suggests that the use of cross-species microarrays in the vesicomyid symbionts may be particularly informative. Affymetrix microarrays based on the *R. magnifica *genome were constructed and hybridized to genomic DNA from two other related vesicomyid symbionts, those of *Vesicomya *sp. mt-II and *Calyptogena kilmeri*. *Vesicomya *sp. mt-II is part of a cryptic species complex which includes the clam formerly known as *Calytogena pacifica *[40].

**Figure 1:**
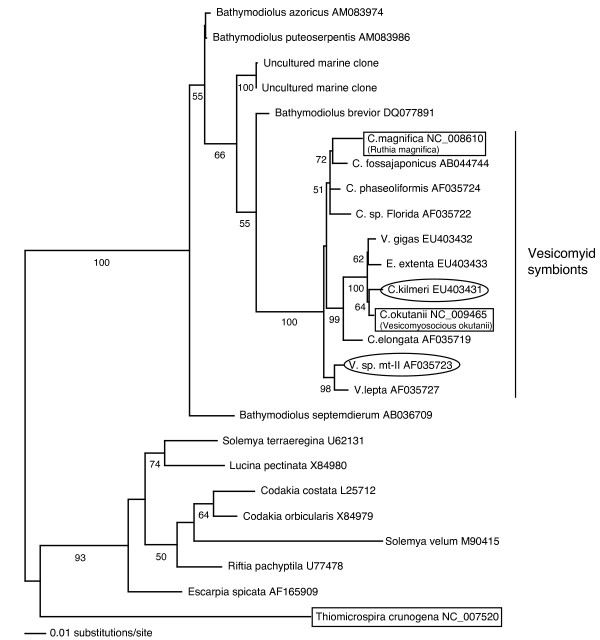
**Molecular phylogeny of chemosynthetic symbionts based on 16S rRNA gene sequences.** These uncultured symbiont taxa are represented by their hosts' scientific name and symbionts, where named, are included in parentheses. A Maximum Likelihood analysis (GTR + gamma) was used with 1,000 bootstrap replicates. Bootstrap values greater than 50% are shown at nodes. Sequenced bacterial genomes in this analysis are boxed while those included in heterologous microarray analyses are circled. *Thiomicrospira crunogena*, a free-living chemoautotroph used as a point of reference for the genomic comparisons, is also highlighted. V = Vesicomya; C = Calyptogena; E = Ectenagena.

A sampling strategy was chosen to illuminate possible effects of phylogenetic relationships and host geochemical environment on vesicomyid symbiont genome evolution. These four symbionts include representatives from each major clade of the vesicomyid symbiont lineage (Figure 1) and therefore allow us to estimate a vesicomyid symbiont core genome. The symbionts investigated here also live within hosts inhabiting two distinct chemosynthetic environments: cold seeps and hydrothermal vents. These habitats differ in their geochemistry with regards to the quantity of oxygen, nitrate and redox state of sulfur available for the symbioses. The data presented here suggest a tremendous amount of genomic stasis and conserved gene content in the vesicomyid symbiont lineage; in the 50–100 Ma that the symbionts have been host associated, their genomes have changed surprisingly little. These data also support existing evidence for lateral acquisition of the vesicomyid symbionts [31]. These results underscore the importance of the symbionts' chemoautotrophic metabolism within their hosts; they emphasize the extent to which host metabolic needs have contributed to genomic evolution in this endosymbiont lineage.

## Results and discussion

Four chemosynthetic symbiont genomes were compared in this study, two sequenced genomes (the hydrothermal vent clam symbiont *Ruthia magnifica *and the cold seep clam symbiont *Vesicomyosocious okutanii*) and gene content for two other strains (the symbionts from the cold seep clam *Calyptogena kilmeri *and the hydrothermal vent clam *V. *sp. mt-II) based on heterologous microarray hybridizations (Figure 1). The *R. magnifica *genome was used to build microarrays for heterologous hybridization to other vesicomyid symbionts. Heterologous microarray hybridization is especially useful when comparing strain-level variation as arrays designed based on one of the strains are likely to hybridize to the DNA of other strains [41-44]. Below, differences between the sequenced genomes are first discussed with reference to a free-living chemoautotroph, *Thiomicrospira crunogena. *Although *T. crunogena *is not part of the direct lineage of the vesicomyid symbionts, it is the closest sequenced relative of the chemosynthetic symbionts and provides us with some perspective as to the gene content necessary to be a functional chemoautotroph. We then discuss chemosynthetic symbiont metabolism and evolution based on both sequence and microarray analyses.

As expected based on their maternal transmission, when compared to *T. crunogena *[38], these symbiont genomes are reduced in size and G + C content (Table 1). The vesicomyid symbiont genomes are about half the size of *T. crunogena's *genome; of the 2,193 proteins in the *T. crunogena *genome, the symbionts encode ~40%. The *T. crunogena *genome encodes enzymes and structures lacking from many intracellular bacteria (flagellar apparatuses, pili, extrachromosomal elements) but many of *T. crunogena's *metabolic capabilities are found in the vesicomyid symbiont genomes (Table 2). Indeed, there are proteins unique to the vesicomyid symbionts when compared to *T. crunogena *and a few of these may increase the symbionts' functional potential within their hosts. For example, the symbiont sulfur oxidation pathway includes both the Sox (sulfur oxidation) and the Dsr (dissimilatory sulfite reductase) enzymes [33], and therefore appears to be more complex than that of *T. crunogena*, which encodes the Sox system exclusively [38]. Thus, these symbiont genomes encode for many of the metabolic pathways of free-living chemoautotrophs despite being reduced in size.

**Table 1:** Genome properties of *Ruthia magnifica *and *Vesicomyososious okutanii *(vesicomyid symbionts) and *Thiomicrospira crunogena *(free-living chemoautotroph).

	Size (Mb)	G+C content (%)	Protein coding (#)	*Coding (%)	rRNA operons (#)
*R. magnifica*	1.2	34.0	976	81	1
*V. okutanii*	1.0	31.6	939	86	1
*T. crunogena*	2.4	43.1	2191	89	3

*percentage of the genome predicted to encode proteins

The sequenced vesicomyid symbiont genomes are quite similar to each other in both gene content and order. Of 976 and 939 proteins encoded in the *R. magnifica *and *V. okutanii *genomes respectively, 886 orthologs are conserved across both, which share a relatively high (82.5%) amino acid identity. The *V. okutanii *and *R. magnifica *genomes also share an extraordinary amount of synteny, as clear from a LAGAN analysis by Kuwahara et al., 2008. Here, an analysis of synteny using MUMmer revealed that a total of 82% of genes in *R. magnifica *remain in the same genomic context and relative location in *V. okutanii*, with a single inversion [39]. This 22.9 kb inversion is comprised of 14 genes including those involved in cofactor biosynthesis (*coaD*, octaprenyl-diphosphate synthase), potassium uptake (*trkHA*), regulation of nitrogen utilization (*ntrXY*), and chaperonins (*dnaKJ*) (see Additional file 1). The inversion did not truncate any of the genes in the region and their orientation does not seem to obviously affect gene function as no operons are disrupted.

There is a striking similarity between these two vesicomyid symbiont genomes, but a few substantial differences stand out. Genes unique to *R. magnifica *and *V. okutanii *are largely in the cell envelope and energy metabolism role categories, respectively (see Additional file 1). The *R. magnifica *genome has a large region comprised of 20 open reading frames lacking in *V. okutanii's *genome (see Additional file 1). This region primarily encodes proteins predicted to be involved in the biosynthesis of polysaccharides and peptidoglycan, components of the cell envelope. The lack of this region in *V. okutanii *suggests that perhaps the vesicomyid symbiont intracellular lifestyle does not require the synthesis of peptidoglycan. Conversely, the *R. magnifica *genome lacks *V. okutanii's *dissimilatory nitrate reductase operon (see Additional file 1), which encodes energy metabolism proteins similar to the membrane-bound, respiratory nitrate reductase (NarGHIJ) found in *Escherichia coli *and other proteobacteria [34,45]. This operon may enable *V. okutanii *to utilize nitrate as a terminal electron acceptor and may reduce competition between host and symbiont for oxygen, although this has yet to be demonstrated. This ability – which may have significant implications for the association's capacity to exploit hypoxic niches – may represent a significant functional difference between these two symbionts.

**Figure 2:**
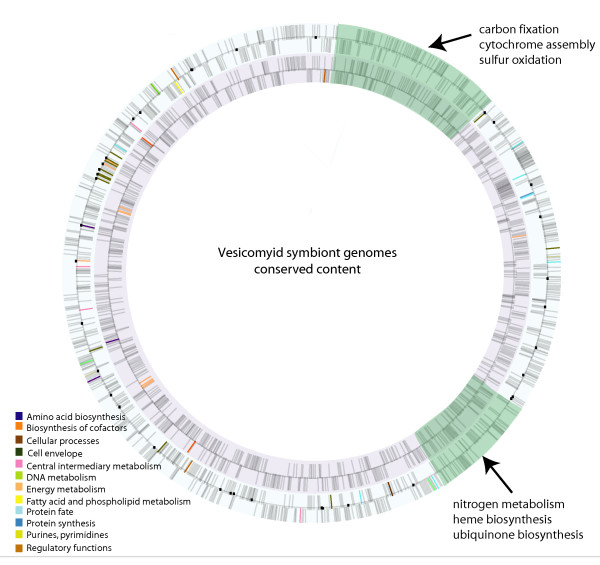
**Differences in functional genomic content between the vesicomyid symbionts.** The circular representations of the sequenced genomes of *Ruthia magnifica *(outer) and *Vesicomyosocious okutanii *(inner) are shown with functional differences between the symbionts colored based on role category. The *C. kilmeri *symbiont genome hybridized to all *R. magnifica *features and therefore is predicted to encode at least the genomic repertoire of *R. magnifica. *Genes putatively absent from the *V. *sp. mt-II symbiont genome based on hybridization to the *R. magnifica *microarray are marked in black. Regions conserved across all four symbiont genomes are highlighted in green.

The heterologous microarray hybridizations of *V. *sp mt-II and *C. kilmeri *symbiont genomic DNA to the *R. magnifica *array confirm the genomic stasis suggested by the sequence comparisons with *V. okutanii. *Indeed, all of the microarray features hybridized to the *C. kilmeri *symbiont gDNA, indicating that this symbiont contains at least the genomic repertoire of *R. magnifica *(Table 2, Figure 2), while 92% of probed genes did not hybridize to the *V. *sp. mt-II gDNA (See Additional file 1). Of this 8.1% of proteins putatively absent from the *V. *sp. mt-II symbiont genome, the majority (34/84) are of unknown function and it is therefore difficult to predict how symbiont metabolism and host interaction might be affected. A total of 854 proteins are shared by all four vesicomyid symbiont genomes and this core genome encodes pathways for sulfur oxidation, nitrogen assimilation and carbon fixation, as well as synthesizing the 19 amino acids and 9 vitamins and cofactors, pathways central to their chemoautotrophic metabolism.

**Table 2:** Number of genes dedicated to each role category in the vesicomyid symbionts.

Role Category	*T. crunogena*	*V. okutanii*	*R. magnifica*	*C. kilmeri	*V. sp. mt-II	**Total conserved
Amino acid biosynthesis	92	91	92	92	91	88
Biosynthesis of cofactors, prosthetic groups, and carriers	100	109	107	107	103	101
Cellular processes	185	49	50	50	48	46
Cell envelope	145	64	79	79	63	58
Central intermediary metabolism	78	50	54	54	53	50
DNA metabolism	89	67	68	68	64	64
Energy metabolism	199	181	176	176	172	167
Fatty acid and phospholipids metabolism	37	26	27	27	27	26
Mobile and extrachromosomal element functions	32	0	0	0	0	0
Protein synthesis	141	122	124	124	118	115
Protein fate	130	81	88	88	88	74
Purines, pyrimidines, nucleotides and nucleosides	48	42	42	42	40	40
Regulatory functions	109	24	26	26	24	22
Signal transduction	19	1	1	1	1	1
Transcription	41	31	31	31	29	29
Transport and binding proteins	205	82	84	84	82	80

Data based on genomics, microarray hybridization results, and validated by polymerase chain reaction and slot blot DNA hybridizations.

*lower estimates of genomic content based on microarray hybridization results and validation methods.

**total conserved gene content = genes shared by all four vesicomyid symbionts included in this study

Interestingly, neither reducing environment nor phylogenetic position correlated strongly with genomic content. The genomes of the two vent clam symbionts (*R. magnifica *and *V. *sp. mt-II symbionts) did not share any gene content to the exclusion of the two symbionts isolated from seep clams (*C. kilmeri *and *C. okutanii *symbionts) and vice versa. In fact, based on the comparative genomic hybridization results, the *C. kilmeri *seep clam symbiont is more similar to the vent clam symbiont *R. magnifica *than to *V. okutanii*. This is a surprising result as the *C. kilmeri *symbiont clades with *V. okutanii *in 16S rRNA phylogenetic trees (Figure 1). Also, the vent clam symbiont *V. *sp. mt-II seems to share genomic excisions with the seep clam symbiont *V. okutanii *in comparison to *R. magnifica; *the same polysaccharide biosynthesis region absent from *V. okutanii *is also missing from the *V. *sp. mt-II genome. These results are inconsistent with predictions of symbiont genomic content based on strict vertical transmission and instead support a mixed transmission strategy for these bacteria [31]. Occasional lateral transmission events, bringing two distinct bacterial symbionts together in the same host background, would provide the necessary opportunity for recombination in this lineage leading to this observed result: a mosaic of evolutionary histories throughout the genome [32].

These four symbionts share a large fraction of their genomic repertoires, such that the total conserved genomic content within each functional role category remains comparatively high (Table 2). When functional differences between these symbionts are mapped onto a circular representation based on the sequenced chromosomes, two large regions were found to be universally conserved across all four symbiont genomes (Figure 2, highlighted in green). One segment, near the origin of replication, contains nearly all of the enzymes necessary for cytochrome (cbb3-type) biosynthesis and the Calvin-Benson-Bassham cycle (transketolase, pyruvate kinase, phosphoglycerate kinase, glyceraldehydes-3-phosphate dehydrogenase, fructose-bisphosphate aldolase). It also harbors enzymes necessary for sulfur oxidation (sulfate adenylyltransferase, APS reductase, ferredoxin) as well as energy conservation (adenylate kinase, pyrophosphatase). The second segment, at 392,401 bp, encodes genes involved in nitrogen metabolism (3-isopropylmalate dehydratase, asparagines synthase), heme biosynthesis (delta-aminolevulinic acid dehydratase, 2-amino-4-hydroxy-6-hydroxymethyldihydropteridine, pyrophosphokinase, thiamine-monophosphate kinase) and ubiquinone biosynthesis. Thus, many of the genes encoding enzymes thought to be fundamental to the symbioses are well conserved across four vesicomyid symbiont strains.

**Figure 3:**
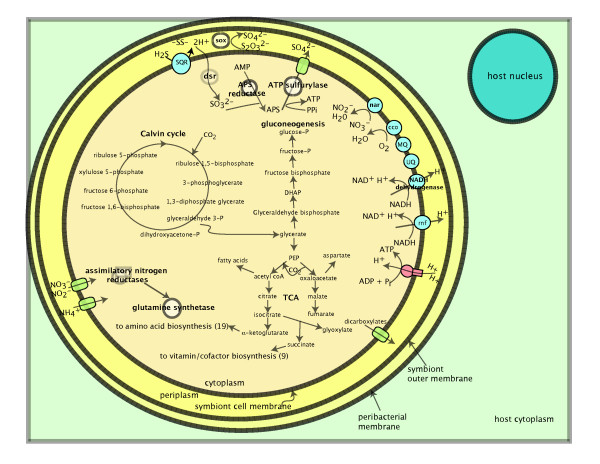
**Hypothetical reconstruction of the vesicomyid last common symbiotic ancestor's (LCSA's) carbon and energy metabolism.** Genes present in at least one of the symbiont lineages examined here are presumed to have been present in the LCSA.

Based on the sequence and microarray data, a reconstruction of the *minimal *gene set for the vesicomyid symbionts' last common symbiotic ancestor (LCSA) is proposed (Figure 3). Genes present in at least one of the symbiont genomes were assumed to have been present in the LCSA. Below, we detail the commonalities and differences between the symbiont genomes with regards to the chemosynthetic metabolisms thought to be important to the symbiosis.

The chemosynthetic symbionts are autotrophic bacteria but there is some question as to whether they are obligately autotrophs or acquire some carbon from the host. The genomic and microarray results from these four vesicomyid symbionts suggest they all have a complete Calvin cycle utilizing a form II RubisCO and that fixed carbon can enter intermediary metabolism as phosphoglyceraldehyde. However, the vesicomyid symbionts intermediary metabolism lacks alpha-ketoglutarate dehydrogenase, a condition thought to be an indicator of obligate autotrophy [46]. The lack of a sugar phosphotransferase (PTS) system in these symbionts plus the lack of organic carbon transporters corroborates this idea. To ameliorate the effects of an incomplete TCA cycle, a glyoxylate bypass is hypothesized for *R. magnifica, V. *sp. mt-II, and *C. kilmeri *symbionts but this key enzyme (isocitrate lyase) is missing from the *V. okutanii *genome [33,34] making regeneration of succinate in *V. okutanii *inexplicable (with the known sequence data). The *V. okutanii *genome is also lacking genes for malate dehydrogenase and fumarate reductase, suggesting significant reduction in comparison to *R. magnifica*. Perhaps *V. okutanii *employs the single citrate transporter (found in all four symbiont genomes) to take up TCA intermediates from the host. This putative sugar transporter (a protein with two SLC13-permease domains and a TrkAC domain) could theoretically function to move TCA cycle intermediates between host and symbiont. Based on this evidence, the LCSA of the vesicomyid symbionts is predicted to have been an obligate autotroph.

However, unlike many free-living obligate autotrophs, the vesicomyid symbionts do not have carboxysomes, polyhedral shaped "organelles" that contain RubisCO and carbonic anhydrase [47,48]. These structures are thought to be involved in dehydration of bicarbonate to provide carbon dioxide (CO_2_) to RubisCO. Although the carboxysome operon seen in other autotrophs is missing from the symbiont genomes, all four vesicomyid symbionts encode putative carbonic anhydrases. It may be that the host bacteriocyte environment maintains a high concentration of CO_2 _limiting the need for carobxysomes (as host respiration is likely to contribute to available inorganic carbon). As is known from the *Riftia *chemosynthetic symbiosis, the host animals might actively trap carbon intracellularly as bicarbonate, limiting the need for carboxysomes in the vesicomyid symbionts [49]. Indeed, the vesicomyid symbionts contain a form II rubisco, the form with low affinity for CO_2_, known from organisms that exist in high concentrations of carbon dioxide [13].

The LCSA for the vesicomyid symbionts is predicted to have derived its energy for carbon dioxide fixation from sulfur oxidation. Sulfur oxidation in all four vesicomyid symbionts is predicted to proceed via the *sox *(sulfur oxidation) and *dsr *(dissimilatory sulfite reductase) genes. However, the *V. okutanii *genome encodes *dsrJNRS *while *R. magnifica's *does not. Because this gene is lacking from the *R. magnifica *genome, it was not queried in the *V. *sp. mt-II or *C. kilmeri *symbiont genomes. While the role of these genes in sulfur oxidation is unclear, they are thought to encode a triheme periplasmic cytochrome (DsrJ), a gene for biosynthesis of siro(heme)amide (DsrN), and cytoplasmic proteins of unknown function (DsrRS) [50,51]. It is therefore unknown how symbiont metabolic function might be affected by the retention of these genes.

Two distinct terminal electron acceptors (a cytochrome c oxidase bc1 complex and a dissimilatory nitrate reductase), are predicted to have been used by the LCSA. Based on sequence data, *R. magnifica *is predicted to rely exclusively on oxygen as a terminal electron acceptor while *V. okutanii *might use either oxygen or nitrate. The *V. okutanii *genome nitrate reductase operon consists of upstream molybdenum cofactor biosynthesis proteins (*moaAD*) followed by *narG and H*, encoding the alpha and beta subunits of nitrate reductase, *narJ*, the delta subunit which inserts the molybdenum cofactor into nitrate reductase, and *narI*, the gamma subunit which is a b-type cytochrome that accepts electrons from quinone to transfer to the alpha subunit [52,53]. When we aligned the *V. okuanii *and *R. magnifica *genomes, we found evidence of this pathway's prior existence in the *R. magnifica *genome. A degenerate pseudogene of the alpha subunit of *V. okutanii's *nitrate reductase sharing 70/122 amino acids remained in the analogous position in the *R. magnifica *genome (see Additional file 1). This evidence suggests that the LCSA was also capable of dissimilatory nitrate reduction. The fact that this pathway has been lost from the *R. magnifica *genome is surprising, given the concentrations of nitrate (40 μM) that exist in deep ocean waters [54].

The loss of the dissimilatory nitrate reductase in *R. magnifica *and retention of the pathway in *V. okutanii *may reflect differences in host geochemical ecology. The *C. okutanii *specimen for the *V. okutanii *genome project was collected off Hatsushima Island, in the Sagami Bay seep sites [34]. The vesicomyid hosts found at Sagami Bay keep their feet buried deep within the silty sediment to access sulfide and may spend the majority of their time in anoxic conditions, where nitrate may be the more abundant oxidant [55]. As the eukaryotic host must use oxygen as a terminal electron acceptor, perhaps the retention of the nitrate reductase in the seep clam symbionts reflects a means of reducing competition for oxygen between host and symbiont. However, it is possible that the amount of oxygen available to the symbiosis in different environments may not translate to distinct microenvironments inside the bacteriocytes; the host hemoglobins [56,57] may somehow buffer the symbionts against extreme anoxic conditions. Further research on the metabolic capabilities of the seep versus vent vesicomyids is needed to determine if ecological differences have contributed to the retention of the dissimilatory nitrate reductases.

A remarkable number of amino acid and cofactor biosynthesis pathways are conserved across all four chemosynthetic symbionts: 19 amino acids and 9 vitamints and cofactors. Although the sequenced symbiont genomes lack the gene encoding homoserine kinase (*thrB*), an enzyme normally utilized in the threonine biosynthetic pathway, there are kinases in all four genomes that could theoretically provide this function. Similarly, only a single cofactor biosynthesis pathway was incomplete; both sequenced symbiont genomes lack the *ubiD/X *gene for ubiquinone biosynthesis from chorismate. Because ubiquinone is required for their energy metabolism, however, it is clear that they either must synthesize this cofactor or obtain it from the host. Thus, the extant vesicomyid symbionts, and therefore their LCSA, are capable of providing all the amino acids and prosthetic groups needed by the host.

Mechanisms for nutrient transport between host and symbiont are not obvious based on the genomic data. Although all four vesicomyid symbionts encode a sec protein export system and the sec-independent Tat system, the use of these pathways for transport of proteins to the host would be energetically costly for the symbiont as the terminal sequences would be wasted with each export. Few known transporters for sugars, amino acids, or vitamin/cofactors were found and instead the symbionts encode ammonium permeases, nitrate/nitrite transporters, and a sulfate exporter; transport mechanisms needed for their chemosynthetic metabolism. Of relevance to the symbiont host interaction is an ABC transporter system found in the *R. magnifica, V. *sp. mt-II and *C. kilmeri *symbiont genomes. This putative hemolysin transporter (*hlyDB, tolC *and a calcium binding hemolysin) is missing in *V. okutanii *without any trace of pseudogenes and, in other organisms, is implicated in pathogenesis [58]. Experimental data are needed to determine what its role may be in the vesicomyid symbionts. The lack of transporters in the sequenced genomes and the high levels of lysozyme in *Calyptogena magnifica *gill tissue [59] has been cited as evidence that the hosts are actively, intracellularly digesting their symbionts. However, many molluscs maintain high levels of lysozyme in their gills as a protective mechanism against pathogenesis [60], and also the reduced peptidoglycan biosynthesis pathways in these genomes would make lysozyme-based digestion unnecessary. It may be that the host utilizes other mechanisms for the digestion of the symbionts such as proteases or reactive oxygen species.

## Conclusion

Symbiosis is a ubiquitous and important ecological strategy for bacteria and eukaryotes, allowing the partners to inhabit environments that neither would be capable of alone. However, for bacterial intracellular symbionts, sequestration within a eukaryotic lineage through vertical transmission can drastically affect symbiont genome evolution, leading to a reduction in gene content and metabolic capabilities. The vesicomyid symbionts may use a mixed strategy for transmission with predominant vertical transmission punctuated by occasional lateral acquisition events [31]. These rare events may give the vesicomyid symbionts the opportunity for recombination and horizontal gene transfer, allowing them to reacquire genes lost through genome reduction [32]. Indeed, in contrast to other sequenced symbiont genomes, the chemosynthetic symbionts of vesicomyid clams have relatively large chromosomes with an extraordinary amount of encoded metabolic capability. Based on genome sizes of free-living autotrophs (~2 Mb in size), the genome of the vesicomyid clam symbionts is only reduced by half. Proteins shared by all four vesicomyid symbiont genomes (which are referred to as the core genome) include the complete pathways necessary for chemoautotrophic metabolism. As is clear from analyses between *V. okutanii *and *R. magnifica*, the symbionts also share a large extent of conserved synteny. Few functional differences were detected in the comparative genomic analysis; indeed the only difference likely to have ramifications for the symbiont metabolism is the ability to use nitrate as an electron acceptor. The last common symbiotic ancestor is predicted to have been an obligate chemoautotroph, utilizing either nitrate or oxygen for the oxidation of reduced sulfur compounds.

Invertebrates are able to thrive at hydrothermal vents due to the metabolic capabilities of their symbionts. The vesicomyids, and indeed many hosts of chemosynthetic bacteria, have evolved small guts and reduced feeding mechanisms and rely primarily on their symbionts for carbon and other nutrients. The data presented here suggest a tremendous degree of conserved gene content in the vesicomyid symbiont lineage, as these symbioses date to 50–100 MYA. This great extent of host dependency may be pressuring symbionts to retain the necessary metabolic pathways needed by the host.

## Methods

### Data deposition

Microarray hybridizations can be found at the Gene Expresion Omnibus (GEO) database (GSE13447). All PCR primers used for validation of these microarray results are attached in Additional file 1.

### Strains

Comparative genomic analyses were conducted using the genome sequences from the hydrothermal vent clam symbiont *Ruthia magnifica *(GenBank:CP000488), the cold seep symbiont *Vesicomyososious okutanii *(GenBank:AP009247), and *Thiomicrospira crunogena *(GenBank:CP000109) a free-living, gamma-proteobacterial, sulfur-oxidizing chemoautotroph. Additionally, heterologous hybridization to an *R. magnifica *microarray was evaluated for two additional symbionts: the symbionts of *Calyptogena kilmeri *from a Monterey Bay cold seep and the symA_VII _phylotype of *Vesicomya *sp. mt-II clams from deep sea vents on the Juan de Fuca Ridge (Goffredi et al., 2003, Stewart et al., 2008).

### Isolation of genomic DNA

Three *Vesicomya *sp. mt-II clams were collected from a hydrothermal vent field on the North Endeavor segment of the Juan de Fuca ridge (47°57.4'N, 129°05.9'W) using the submersible *Alvin *(dive 2413, depth 2200 m). Three *C. kilmeri *clams were collected using the ROV Tiburon (depth 970 m) from the Montery Canyon (36°46.53'N, 122°5.21'W). Symbiont-containing gills were dissected out of the clams, frozen in liquid nitrogen, and kept at -80°C until processed. Thawed tissue was treated with DNase (0.8 mg/ml, 50°C for 1.5 hr with gentle agitation) to remove host DNA from the samples. This DNase treatment was optimized and relative quantities of host and symbiont DNA determined by slot blot hybridizations using universal 16S (GCT GCC TCC CGT AGG AGT) and 18S (GCA ATA ACA GGT CTG TGA TGC CC) rRNA probes. Although not quantitative, a qualitative estimate of enrichment of symbiont DNA was achieved. Tissues were ground in liquid nitrogen, placed in lysis buffer (20 mM EDTA, 10 mM Tris-HCl, pH 7.9, 0.5 mg/ml lysozyme, 1% Triton X-100, 500 mM guanidine-HCl, 200 mM NaCl) and kept at 40°C for 2 hr. After subsequent RNase and proteinase K treatments, the samples were centrifuged and the supernatant loaded onto a Qiagen genomic tip column and processed according to manufacturer's instructions.

### Microarray construction

NimbleExpress Probe Arrays with probes representing all 1022 open reading frames in the *Ruthia magnifica *genome were produced by Affymetrix (manufactured in the 49 format) and were synthesized with approximately 20 oligonucleotide probes (25-mers) per putative transcript and 10–20 probe pairs within a probe set. For the design of the *R. magnifica *arrays, we provided Affymetrix with partial host genome sequence to further limit the probability of host hybridization to the symbiont array. Each probe pair contains a sequence complementary to the target sequence (PM) and a sequence with a mismatch in position 13 (MM). The ratio of PM/MM hybridization of target sequence in each probe pair over the entire probe set is used to call expression levels. It should be emphasized that because *R. magnifica *was used as the reference strain for array design, genes not found in its genome were not queried in the *Vesicomya *sp. mt-II and *C. kilmeri *symbiont genomes. Also, low hybridization to the *R. magnifica *features suggests that either the genes are absent in the target sequence or are sufficiently divergent to prevent hybridization.

### Microarray Hybridization

The DNA was prepared for biotin end-labeling and hybridization by DNase treatment (0.5 U per 10 μg DNA in 100 μl for 15 min at 25 C). After validation of fragment sizes (50–100 bp) via gel electrophoresis, DNA was precipitated with ethanol and labeled for one hour using terminal deoxynucleotide transferase and biotin-ddUTP (Enzo BioArray). The reactions were used directly for Affymetrix hybridization. Labeled gDNA targets were hybridized to these arrays using the ProkGE_WS2v3 fluidics protocol on the GeneChip 400 Fluidics Station (Affymetrix, Inc). Hybridization cocktails were assembled using the GeneChip reagents and contained 100 μl of 2× hybridization mix, 2.5 μl of oligo B2, 10 μl of 20× controls and 6 μg of labeled target. As positive controls, *Bacillus subtilus *DNA clones were spiked into our cocktails. Each experiment (*Vesicomya *sp. mt-II symbiont, *C. kilmeri *symbiont) was performed in triplicate with at least 2 biological replicates (two distinct clam individuals).

### Microarray Data Analysis

Hybridization intensity data were extracted from the array images and scaled universally across all experiments and normalized using Resolver microarray software (Rosetta Syllego). Values derived from the hybridization of *R. magnifica *gDNA to the arrays were used as a baseline for the genomic analyses. Absence/presence of the *R. magnifica *homologs in *C. kilmeri *and *Vesicomya *sp. mt-II were determined by comparing hybridization signals between the baseline and the two strains. Probe sets with a ratio of >0.25 were considered present, and those with a ratio of <0.25 were considered absent or excessively divergent.

### Verification of the microarray hybridization thresholds

The use of these hybridization intensity thresholds was validated using PCR and slot blot nucleotide hybridizations. We randomly chose 20 genes, and primers were designed (see Additional file 1) based on the *R. magnifica *and *V. okutanii *genomes and bands were generated (data not shown). Five genes called as "absent/divergent" were also targeted for amplification but could not be amplified (data not shown). We also selectively targeted the polysaccharide biosynthesis region from *R. magnifica *(absent in *V. okutanii *and *V. *sp. mt-II symbiont) using slot blot hybridizations. A probe was designed to target the o-antigen polymerase gene from *R. magnifica *for hybridization against *V. *sp. mt-II and *C. kilmeri *symbiont gDNA. *R. magnifica, V. *sp. mt-II symbiont, and *C. kilmeri *symbiont whole genomic DNA extracts were denatured (0.4 M NaOH, 10 mM EDTA, 10 mins at 100°C) before loading onto the Bio-Dot SF Microfiltration Apparatus (Biorad) and Zeta-probe mebrane (Biorad). After applying vacuum, the membrane was washed in 2 × SSC and DNA was crosslinked using the UV Stratalinker 2400 (Stratagene). After prehybridization at 30°C with ExpressHyb (Clonetech) hybridization buffer, radioactively (γ-32P-ddATP) end-labeled 300 bp fragements (T4 PNK) were hybridized to the membrane overnight at 42°C. The membranes were then washed at 25°C three times in 50 mL of 2 × SSC, 0.1% SDS and once in 0.1 × SSC, 0.5% SDS before exposing film for 2 hours and subsequent development.

### Bioinformatics

To find putative orthologous proteins between the sequenced genomes, the program RSD (Reciprocal Smallest Distance algorithm) [61] was used to compare the *R. magnifica *genome to that of the *V. okutanii *genome. A 1e^-3 ^cutoff for the significance threshold and an alignment length threshold of 80% were used which yielded a total of 858 conserved orthologous proteins. We then used reciprocal BLAST to identify another 28 orthologous proteins. The MUMmer 3 software package [62] was used for analysis of synteny and the BLAST program [63] was used to indentify percent identity and similarity between the conserved proteins.

## Authors' contributions

ILGN conceived of the experiment, generated and analyzed the microarray and comparative sequence data, and wrote the manuscript. PRG provided animal samples, substantial intellectual contributions to the experimental design, and contributed to production of the manuscript. CMC procured the funding for this project, provided supervision to ILGN, and contributed to the production of the manuscript. All authors have read and approve of this final manuscript.

## Supplementary Material

Additional file 1 **Supplementary tables and figures for "Comparative Genomics of Chemosynthetic Symbionts".** Schematic representation of genomic comparisons between *V. okutanii *and *R. magnifica. *Also included are tables listing unique gene content in the *V. okutanii *and *R. magnifica *comparisson as well as tables of microarray data from the hybridization of *V. *sp. mt-II symbiont to the *R. magnifica *array. Finally, a list of primers used to validate the microarray data are included. Click here for file
