# Supplementary material for: Comparative genomics of vesicomyid clam (Bivalvia: Mollusca) chemosynthetic symbionts
Source: BMC Genomics. 2008 Dec 4;9:585. doi: 10.1186/1471-2164-9-585 (PMC2642828; doi:10.1186/1471-2164-9-585)
Supplement: Additional file 1 — Supplementary tables and figures for "Comparative Genomics of Chemosynthetic Symbionts". Schematic representation of genomic comparisons between V. okutanii and R. magnifica. Also included are tables listing unique gene content in the V. okutanii and R. magnifica comparisson as well as tables of microarray data from the hybridization of V. sp. mt-II symbiont to the R. magnifica array. Finally, a list of primers used to validate the microarray data are included. [file 1471-2164-9-585-S1.pdf]

Supplementary material for Newton *et al.* manuscript
"Comparative genomics of Vesicomyid clam (Bivalvia:Mollusca) Chemosynthetic Symbionts"

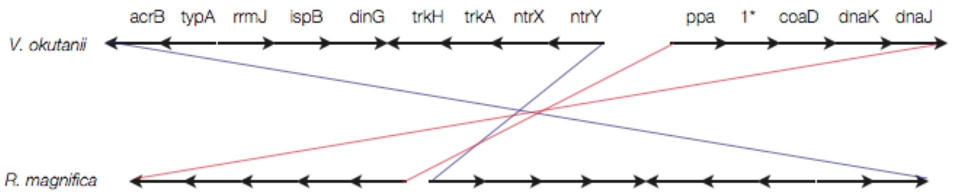

Supplementary Figure 1. The single inversion found in the genomic comparison between *Vesicomyosocious okutanii* (COSY_0334-COSY_0347) and *Ruthia magnifica* (Rmag_0369-0352). Genes in the inversion encode an acriflavin resistance protein (acrB), a stress adaptation protein (typA), an RNA methyltransferase (rrmJ), isoprenoid quinone biosynthesis protein (ispB), a DNA helicase (dinG), potassium uptake transporters (trkH, trkA), a two compontent sensor/regulator system (ntrX, ntrY), (ppa), a hypothetical protein (1*), a coenzyme biosynthesis protein (coaD), an initiator of chromosomal replication (dnaK), and a molecular chaperone (dnaJ).

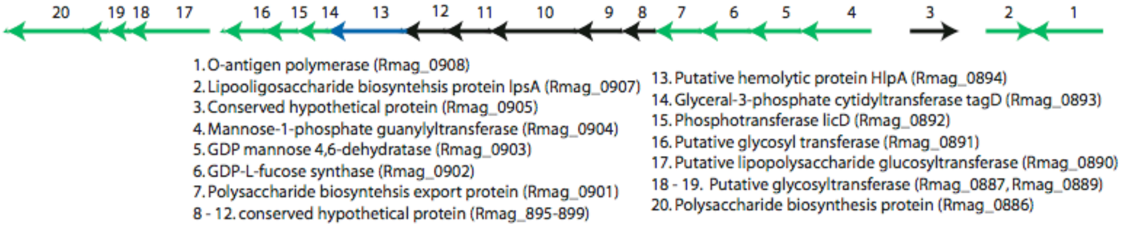

Supplementary Figure 2. Polysaccharide biosynthesis region missing from the *Vesicomyosocious okutanii* genome and putatively absent from the *Vesicomya* sp. mt-II symbiont based on microarray hybridization and PCR and slot blot validation. Hypothetical proteins are colored black.

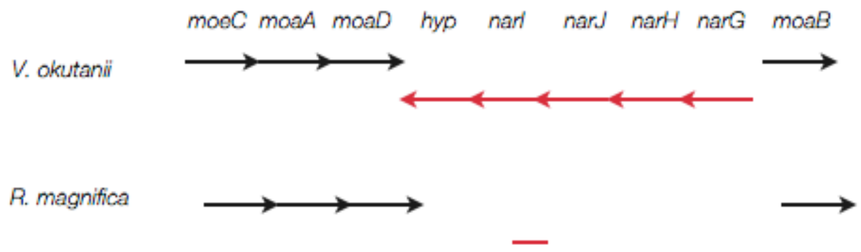

Supplementary Figure 3. Genes encoding the respiratory nitrate reductase in *Vesicomyosocious okutanii* (COSY_0642-COSY_0650) as compared to the same genomic region in *Ruthia magnifica* (Rmag_0692 – Rmag_0698). The pseudogene fragment of the alpha-subunit *narI,* found in the *R. magnifica* genome is shown as a red bar beneath its location in *V. okutanii's* genome. *Hyp* = hypothetical protein.

Supplementary Table 1. Gene content with a defined function unique to the *Ruthia magnifica* and *Vesicomyososious okutanii* genomes.

Vesicomyososious okutanii	Functional Role Category	Locus number
Cobalamin dependent methionine synthase I	Aminoacid biosynthesis	COSY_0466
Alpha-ribazole phosphatase	Biosynthesis of cofactors, prosthetic groups, and carriers	COSY_0841
Cobalamin adenosyltransferase	Biosynthesis of cofactors, prosthetic groups, and carriers	COSY_0583
L-asparaginase	Energy metabolism	COSY_0226
Nitrate reductase alpha subunit	Respiratory nitrate reductase	COSY_0649
Nitrate reductase delta subunit	Respiratory nitrate reductase	COSY_0647
Nitrate reductase beta subunit	Respiratory nitrate reductase	COSY_0648
Nitrate reductase gamma subunit	Respiratory nitrate reductase	COSY_0646
dsrR	Sulfur oxidation	COSY_0782
dsrN	Sulfur oxidation	COSY_0783
dsrJ	Sulfur oxidation	COSY_0786
Stringent starvation protein B	Regulatory functions	COSY_0008
Ruthia magnifica		
Cobalamin independent methionine synthase, metE	Aminoacid biosynthesis	Rmag_0985
Sodium pump decarboxylases, gamma subunit	Aminoacid biosynthesis	Rmag_0849
Cell division protein, FtsA	Cell division	Rmag_0443
Septum formation initiator	Cell division	Rmag_0754
3-deoxy-manno-octulosonate cytidyltransferase	Cell envelope	Rmag_0841
GDP-mannose 4,6-dehydratase	Cell envelope	Rmag_0903
Glycosyl transferase, family 2	Cell envelope	Rmag_0891
Glycosyl transferase, family 51	Cell envelope	Rmag_0663
Glycosyl transferase, group 1	Cell envelope	Rmag_0890
Gycosyl hydrolase, BNR repeat-containing	Cell envelope	Rmag_0251
Lytic transglycosylase, catalytic	Cell envelope	Rmag_0762
Mannose-1-phosphate guanylyltransferase/mannose-6-phosphate isomerase	Cell envelope	Rmag_0904
Membrane bound O-acyl transferase,	Cell envelope	Rmag_0219

MBOAT family		
Multiple antibiotic resistance (MarC) related protein	Cell envelope	Rmag_0519
O-antigen polymerase	Cell envelope	Rmag_0908
Polysaccharide biosynthesis protein CapD	Cell envelope	Rmag_0886
Polysaccharide biosynthesis protein	Cell envelope	Rmag_0901
Sugar transferase	Cell envelope	Rmag_0887
N-acetylmuramyl-L-alanine amidase	Cell envelope	Rmag_1042
Isocitrate lyase	Central intermediary metabolism	Rmag_0538
Sulfatase	Central intermediary metabolism	Rmag_0937
Fumarate reductase	Central intermediary metabolism	Rmag_0966
Malate dehydrogenase	Central intermediary metabolism	Rmag_0819
Helix-turn-helix, Fis-type	DNA metabolism	Rmag_0968
Glucose-1-phosphate adenylyltransferase related protein	Energy metabolism	Rmag_0898
Glycine dehydrogenase	Energy metabolism	Rmag_0821
NAD-dependent epimerase/dehydratase	Energy metabolism	Rmag_0902
Diacylglycerol kinase, dgkA	Fatty acid and phospholipids biosynthesis	Rmag_0983
Disulfide bond isomerase, DsbC	Protein fate	Rmag_0210
Aminopeptidase N	Protein fate	Rmag_0440
ATP-dependent Clp protease adaptor protein, ClpS	Protein fate	Rmag_0912
Heat shock protein, Hsp90	Protein fate	Rmag_0493
Methionine-R-sulfoxide reductase, msrB	Protein fate	Rmag_0196
Protein tyrosine phosphatase	Protein fate	Rmag_0159
Secretion protein HlyD	Protein fate	Rmag_0256
Sun protein	Protein synthesis	Rmag_0209
Ribonucleotide reductase regulator NrdR-like	Regulatory functions	Rmag_0631
Transcriptional regulator, LysR family	Regulatory functions	Rmag_0986
Aminoglycoside phosphotransferase	Toxin production and resistance	Rmag_0468
MscS mechanosensitive ion channel	Transport and binding	Rmag_0742
Outer membrane efflux protein	Transport and binding	Rmag_0253

Supplementary Table 2. Hybridization intensity ratios for the heterologous hybridizations of genomic DNA from *Vesicomya* sp. *mt-II* and *Calyptogena kilmeri* symbionts to the *R. magnifica* microarrays. Shown are postscanning and normalization ratios of values < 0.25 in the *V.* sp. mt-II hybridization suggesting absence of this gene.

***V.* sp. mt-II**	***C. kilmeri***	**Gene**	**Functional Role Category**
0.12983	0.73354	fructosamine kinase	Aminoacid biosynthesis
0.13175	0.43383	Uroporphyrinogen III synthase, hemD	Biosynthesis of cofactors, prosethetic groups, and carriers
0.08403	0.81611	FolC bifunctional protein	Biosynthesis of cofactors, prosthetic groups and carriers
0.16835	1.02705	Riboflavin synthase	Biosynthesis of cofactors, prosthetic groups, and carriers
0.16291	0.71456	tRNA(Ile)-lysidine synthetase	Biosynthesis of cofactors, prosthetic groups, and carriers
0.12293	0.4746	ATP-dependent metalloprotease FtsH	Cell division
0.16708	0.87893	Putative multicopper oxidase SufI	Cell division
0.12802	0.5945	glucose-1-phosphate adenylyltransferase related protein	Cell envelope
0.17749	0.76326	glycerol 3-phosphate cytidyltransferase	Cell envelope
0.11894	0.42822	glycosyl transferase, family 2	Cell envelope
0.21953	0.5988	glycosyl transferase, family 2	Cell envelope
0.20508	0.7405	glycosyl transferase, family 51	Cell envelope
0.12832	0.37082	glycosyl transferase, group 1	Cell envelope
0.22116	0.51705	lipid A biosynthesis acyltransferase, htrB	Cell envelope
0.15057	0.45693	mannose-1-phosphate guanylyltransferase/mannose-6-phosphate isomerase	Cell envelope
0.2303	0.55607	membrane bound O-acyl transferase, MBOAT family protein	Cell envelope
0.23713	0.52534	O-antigen polymerase	Cell envelope
0.11704	0.56629	Phosphomannomutase	Cell envelope
0.17159	0.55429	polysaccharide biosynthesis protein	Cell envelope

0.14084	0.41958	polysaccharide biosynthesis protein CapD	Cell envelope
0.12984	0.46277	putative 3-deoxy-D-manno-octulosonate 8-phosphate phosphatase	Cell envelope
0.20452	0.4394	sugar transferase	Cell envelope
0.20142	0.6077	UDP-N-acetylglucosamine 1-carboxyvinyltransferase, murA	Cell envelope
0.16766	0.5665	putative sulfatase	Central intermediary metabolism
0.14364	0.46483	chromosomal replication initiator protein DnaA	DNA metabolism
0.24375	0.66967	helix-turn-helix, Fis-type	DNA metabolism
0.24311	0.51707	non-canonical purine NTP pyrophosphatase, rdgB/HAM1 family	DNA metabolism
0.20671	0.79926	transcription-repair coupling factor, mfd	DNA metabolism
0.15537	0.41747	cytochrome c4 precursor	Energy metabolism
0.17538	0.48402	electron transport protein SCO1/SenC	Energy metabolism
0.16408	0.63032	glycine dehydrogenase	Energy metabolism
0.19248	0.69157	pyridine nucleotide-disulphide oxidoreductase dimerisation region	Energy metabolism
0.24385	0.75608	glutamyl-tRNA(Gln) amidotransferase, C subunit	Protein synthesis
0.08394	0.31908	Glycine--tRNA ligase	Protein synthesis
0.17975	0.34617	histidyl-tRNA synthetase	Protein synthesis
0.16935	0.66401	lysyl-tRNA synthetase	Protein synthesis
0.18665	0.55875	ribosomal protein S1	Protein synthesis
0.18458	0.87695	tRNA (guanine-N(7)-)-methyltransferase	Protein synthesis
0.11343	0.39508	phosphoribosylglycinamide formyltransferase	Purines, pyrimidines, nucleosides, and nucleotides
0.20773	0.63945	putative dUTPase	Purines, pyrimidines, nucleosides, and nucleotides
0.17186	0.51668	periplasmic sensor signal transduction histidine kinase	Regulatory functions
0.1888	0.64176	response regulator receiver protein	Regulatory functions
0.21847	0.48747	ribonuclease T	Transcription
0.19138	0.63069	DEAD/DEAH box helicase domain protein	Transcription
0.22216	0.47717	ATPase AAA-2 domain protein	Transport and binding proteins

0.21999	0.56583	major facilitator superfamily transporter	Transport and binding proteins
0.18481	0.98867	FxsA cytoplasmic membrane protein	Unknown
0.09074	0.47415	histidine triad (HIT) protein	Unknown
0.23639	0.41234	hypothetical protein Rmag_0019	Unknown
0.1776	0.70103	hypothetical protein Rmag_0095	Unknown
0.22958	0.55432	hypothetical protein Rmag_0128	Unknown
0.10457	0.47824	hypothetical protein Rmag_0130	Unknown
0.20764	0.55323	hypothetical protein Rmag_0135	Unknown
0.19832	0.5275	hypothetical protein Rmag_0199	Unknown
0.15063	0.51767	hypothetical protein Rmag_0252	Unknown
0.22456	0.59805	hypothetical protein Rmag_0376	Unknown
0.16268	0.61274	hypothetical protein Rmag_0586	Unknown
0.22174	0.70979	hypothetical protein Rmag_0588	Unknown
0.06919	0.76003	hypothetical protein Rmag_0637	Unknown
0.23634	0.76323	hypothetical protein Rmag_0710	Unknown
0.15132	0.547	hypothetical protein Rmag_0771	Unknown
0.13676	0.37365	hypothetical protein Rmag_0776	Unknown
0.11596	0.68398	hypothetical protein Rmag_0782	Unknown
0.14521	0.72345	hypothetical protein Rmag_0782	Unknown
0.15191	0.55317	hypothetical protein Rmag_0802	Unknown
0.22014	0.62579	hypothetical protein Rmag_0829	Unknown
0.1937	0.78476	hypothetical protein Rmag_0892	Unknown
0.23401	1.32977	hypothetical protein Rmag_0895	Unknown
0.19303	0.68474	hypothetical protein Rmag_0896	Unknown
0.06688	0.38596	hypothetical protein Rmag_0897	Unknown
0.14871	0.67353	hypothetical protein Rmag_0899	Unknown
0.10478	0.50188	hypothetical protein Rmag_0945	Unknown
0.15238	0.79183	hypothetical protein Rmag_1056	Unknown
0.07887	0.42999	methyltransferase FkbM	Unknown
0.2127	1.07755	protein of unknown function DUF1111	Unknown
0.16893	0.48027	protein of unknown function DUF423	Unknown
0.10283	0.58761	protein of unknown function DUF45	Unknown
0.11658	0.45264	protein of unknown function DUF461	Unknown
0.15547	0.32972	protein of unknown function UPF0118	Unknown
0.17119	0.6746	Redoxin domain protein	Unknown

Below are primers used in the study "Comparative genomics of Vesicomyid clam (Bivalvia:Mollusca) chemosynthetic symbionts"

**Amplicon annotation**	**No. Bases**	**Primer Sequence**	**Tm** (50mM NaCl)
Primers used to generate probe for the dissimilatory nitrate reductase operon			
nitrate_red Set 1 Forward Prim	24	TCA ACA CCT TGA CGT GAG GTC CAT	60.242
nitrate_red Set 1 Reverse Prim	24	AAG CAG TAG ATC CTG CGT GGG AAT	60.254
Primers used to amplify genes putatively present in the *C. pacifica* and *C. kilmeri* symbiont genomes based on microarray hybridizations			
alanine racemase F	24	TGA AAG CCA ATG CTT ATG GCC ACC	60.333
alanine racemase R	24	ATG GCT GTT GAT TTG TCG GCT GTC	60.077
corproporphyrinogen oxidase F	24	CAG CCA AAC AAG CGT GTG ATC CAT	60.286
corproporphyrinogen oxidase R	24	AAC CTT GCT TCT TCG CTA CCT GGA	60.312
glutamyl tRNA synthetase F	24	CGG TAC AAG CAA GTT GTT CGG CAA	60.252
glutamyl tRNA synthetase R	24	GCA GGC GCC TTA TTG ATG CTC TTT	60.203
protein of unknown function DUF423 F	25	AGC ATT AGG TGG TGC TTT AGT AGT G	56.74
protein of unknown function DUF423 R	29	TCC AAA TAC TTT GAT CGA TAG ACC TAG AC	55.764
thiamine-monophosphate kinase F	24	TAA GTG ATT TGG CAG CAG TGG GTG	59.718
thiamine-monophosphate kinase R	24	CAC CAG CAA GCA CTA AGC ACC AAT	60.131
ribosomal protein L6 F	24	TCA AGG CGT AAC AGA AGG TTG GGA	60.297
ribosomal protein L6 F	24	TGC TCA TCA ATG TAG CAA ACG CCC	60.101
NADH (or F420H2) dehydrogenase, subunit C	24	AGA CTG GAA TGG TAA TGC GAG TGC	59.252
NADH (or F420H2) dehydrogenase, subunit C	24	AAA CCA CCC GAC CTA AGT CTT CGT	60.002
ABC transporter related F	26	GCT GGT ATC GAT AAA CCC ACT CAA GG	59.045
ABC transporter related R	24	ACC AGA AAG CTC ACT TGG TAA GCG	59.345
cytochrome b/b6, N-terminal domain F	24	ATT TGG CTC TGT GCC CGT ATT TGG	60.032
cytochrome b/b6, N-terminal domain R	24	GCC AAC AAC ACC TGG GAA TTG TGA	60.144
hydrolase, TatD family F	24	AGG CGT GCA TCC TTG TGA ACT AGA	60.282
hydrolase, TatD family R	24	TAC TCA CGC CGC GAA TTT CAG CTA	60.497
deoxyxylulose-5-phosphate synthase F	24	CTT AGC GCC CAA TCT TGG CAC AAT	60.223
deoxyxylulose-5-phosphate synthase R	24	GCA TGC TGT TCT GCA ATA CCC ACA	60.155
ribosomal protein S14	28	GTC TAT GAT AAA TAG AGA CAT CAA GCG T	54.524
permease YjgP/YjgQ family protein f	24	TAC GAG CGC CAG ATA CGT TGG ATT	60.195
permease YjgP/YjgQ family protein r	24	CAG CAA TGT TGG TAT TGA GGC GCT	60.136

ribonucleotide reductase regulator NrdR-like F	24 TGA ATG CGT TTC TTG TGG TGA GCG	60.258
ribonucleotide reductase regulator NrdR-like R	24 CTT CCA TTA CCC ACT CGC CAA TCT	58.948
glucose-6-phosphate isomerase f	24 AGG TTC ACT ACT AGC GCA ACA TGC	59.355
glucose-6-phosphate isomerase r	24 TTG CCA GAT TCT ACA CCA GGC TGA	60.205
ribosomal protein L1 F	24 CTT CAA GTG GCT GCA CAA GAA GCA	60.195
ribosomal protein L1 R	24 TCC ACC GTA ATG CCC ATT GAC CTA	60.068
sulfur oxidation protein SoxY-like F	24 ACC GCC ACA ACC ACC AAT TGT TAC	60.274
sulfur oxidation protein SoxY-like R	24 TGC GAT GTC AGG TTC TGT GTT TGC	60.105
o_antigen_poly Set 2 Forward P	24 CCA AAC CAC CAC GCC ACA TTG ATA	59.929
o_antigen_poly Set 2 Reverse P	24 AGG TAT TGA CAG GGT AGA AGC AGG	58.082
citrate transporter Set 2 Forw	24 TCC CAT CTC TTG GAT GTT GGG CAT	60.486
citrate transporter Set 2 Reve	24 TAG TCG CGC CTA CAT TGG ACA TCA	60.03
citrate transporter Set 1 Forw	24 ACC ATG GTG GGT TCT AGC CCT TTA	59.97
citrate transporter Set 1 Reve	24 TGA CCC AAG GTG CCA TAT CTC CAA	60.16

Primers used to amplify genes putatively absent from the *C. pacifica* symbiont genome based on microarray hybridizations

Rmag_0804 Set 1 Reverse Primer	24 CTG TCA TTA AAG CGG CTG AGA CTG	57.859
Rmag_0804 Set 1 Forward Primer	20 AGA ACC TAG GTC CAG CTG TT	55.488
NADdependent epimerase Set 1 F	24 TCT CGC ATT GCT TTA CCT GTA CCC	59.022
NADdependent epimerase Set 1 R	24 AGG CTT AGT CGG TTC AGC AAT CGT	60.446
putative iron Set 2 Forward P	24 CCT GCT ACC AAC GCA CCA TTT GTT	60.28
putative iron Set 2 Reverse P	24 CAT TGC AAC GGC AAA CTA CAC GGA	60.31
serine oacyl Set 1 Forward Pr	24 GCA CCA GCA CCA ATC ACG ACA TTA	59.824
serine oacyl Set 1 Reverse Pr	24 CCT GCT GCC AGA AAC ACC TTT GAA	60.091
oxaloacetatedecar Set 1 Forwar	24 TTA CCT GCT TGT AGC ACT GGC TCA	60.303
oxaloacetatedecar Set 1 Revers	24 GCT GTT GAA GCT GGC ATT GAT CGT	60.266
